# Treatment course and outcomes following drug and alcohol-related traumatic injuries

**DOI:** 10.1186/1752-2897-5-3

**Published:** 2011-01-20

**Authors:** Matthew C Cowperthwaite, Mark G Burnett

**Affiliations:** 1 NeuroTexas Institute at St. David's HealthCare, St. David's Medical Center, 1015 East 32nd Street, Suite 404, Austin, Texas 78705, USA; 2 Center for Systems and Synthetic Biology, The University of Texas at Austin, 1 University Station, A4800, Austin, Texas 78712, USA

## Abstract

**Background:**

Alcohol and drug use is known to be a major factor affecting the incidence of traumatic injury. However, the ways in which immediate pre-injury substance use affects patients' clinical care and outcomes remains unclear. The goal of the present study is to determine the associations between pre-injury use of alcohol or drugs and patient injury severity, hospital course, and clinical outcome.

**Materials and methods:**

This study used more than 200,000 records from the National Trauma Data Bank (NTDB), which is the largest trauma registry in the United States. Incidents in the NTDB were placed into one of four classes: alcohol related, drug related, alcohol-and-drug related, and substance negative. Logistic regression models were used to determine comorbid conditions or treatment complications that were significantly associated with pre-injury substance use. Hospital charges were associated with the presence or absence of drugs and alcohol, and patient outcomes were assessed using discharge disposition as delimited by the NTDB.

**Results:**

The rates of complications arising during treatment were 8.3, 10.9, 9.9 and 8.6 per one hundred incidents in the alcohol related, drug related, alcohol-and-drug related, and substance-negative classes, respectively. Regression models suggested that pre-injury alcohol use is associated with a 15% higher risk of infection, whereas pre-injury drug use is associated with a 30% higher risk of infection. Pre-injury substance use did not appear to significantly impact clinical outcomes following treatment for traumatic injury, however.

**Conclusion:**

This study suggests that pre-injury drug use is associated with a significantly higher complication rate. In particular, infection during hospitalization is a significant risk for both alcohol and drug related trauma visits, and drug-related trauma incidents are associated with increased risk for additional circulatory complications. Although drug and alcohol related trauma incidents are not associated with appreciably worse clinical outcomes, patients experiencing such complications are associated with significantly greater length of stay and higher hospitalization costs. Therefore significant benefits to trauma patients could be gained with enhanced surveillance for pre-injury substance use upon admission to the ED, and closer monitoring for infection or circulatory complications during their period of hospitalization.

## Introduction

Traumatic injury is the most frequent cause of admission to the emergency department (ED) in U.S. hospital systems [1]. The United States Centers for Disease Control (CDC) estimated that 44 million injuries occur each year that require hospital treatment [2]. The cost of medical care for these injuries is approximately $117 billion [2]. Traumatic injuries are therefore a major source of morbidity and mortality in society, and a significant burden on the U.S. healthcare system.

It has been estimated that as many as 50% to 70% of patients with traumatic injuries have detectable levels of alcohol in their blood at the time of admission [3-5]. The frequency of pre-injury drug use in trauma patients has been estimated to be around 40 to 50 percent [6-10]. Alcohol and drug use is therefore known to be a major contributor to the *incidence *of traumatic injury, [3,11-19] however the impact of alcohol and drug use on treatment course and clinical outcomes is less clear.

Previous studies have considered the effects of pre-injury drug and alcohol use on hospital treatment and clinical outcomes [6,10,16,20-34]. Yet, these studies have come to different conclusions about the roles of pre-injury alcohol and drug use in traumatic injury and its subsequent treatment. For example, some studies have suggested that pre-injury substance use is associated with more significant injury, whereas other studies have found no such association. These differences likely result from several factors, such as the cohort of patients included in the study, or whether the study considered acute or chronic substance use. Thus, there remains a need to further understand how pre-injury drug and alcohol use affects the treatment course during hospitalization and subsequent clinical outcomes following traumatic injury.

The present study represents the largest retrospective study yet to consider the impact of immediate pre-injury substance use on hospital course and outcomes following traumatic injury. The goals of this study are to determine if pre-injury substance use is associated with (i) significantly less healthy patient populations, (ii) more severe injuries, (iii) significantly different hospital course, and (iv) significantly worse clinical outcomes.

## Materials and methods

### Dataset

The National Trauma Data Bank (NTDB) is a database maintained by the American College of Surgeons (ACS) [35]. The NTDB accepts voluntary data submissions from participating trauma centers; prior to release the data is curated to ensure the most accurate data possible. The data submissions undergo cleaning to standardize and improve the quality of the data; submissions from hospitals are checked for completeness, logical consistency, and formatting errors. In this study, we used version 7.1 (May 2008) of the NTDB, which contained data describing injury incidents voluntarily submitted by 770 trauma centers in the United States.

Injury incidents were included if and only if we could determine the presence or absence of both drugs and alcohol upon admission to the emergency department (ED). Incidents were excluded if the drug or alcohol status was either not determined or not reported. Injury incidents were placed into one of four classes: (i) "alcohol related" incidents are those in which alcohol use was present and drug use was absent; (ii) "drug related" incidents are those in which drug use was present and alcohol use was absent; (iii) "alcohol-and-drug related" incidents involved pre-injury use of both alcohol and drugs; and, (iv) "substance negative" incidents are those in which there was no reported pre-injury use of drugs or alcohol. These classes were created to distinguish the between effects associated with alcohol and drugs used individually and in combination.

Demographic variables included in the study were age and gender. Age was modeled as a continuous variable, whereas gender was modeled as a dichotomous variable. Clinical variables included injury severity score (ISS), pre-existing comorbidities, secondary complications and discharge disposition (outcome). ISS was modeled as a continuous variable, whereas comorbidities, complications, and outcomes were modeled as dichotomous variables indicating the presence or absence of the condition. All incidents were required to meet the following inclusion criteria: (i) age must be at least 16; (ii) ISS must be between 0 and 75; and (iii) the gender must be delimited either "male" or "female". These criteria primarily check the validity of the data; the age validation ensures that we exclude potential pediatric trauma cases. These checks were performed to ensure the integrity of the data, and thereby increase the accuracy of the primary study variables.

The pre-existing comorbidities contained in the NTDB were used to test whether the substance-abusing trauma population is significantly less healthy than the trauma population as a whole. The comorbid conditions delimited by the NTDB cover many common health conditions, such as diabetes, hypertension, obesity, and depression. Each comorbid condition was tested independently using logistic regression models in which the comorbid condition was modeled as a dichotomous response variable and regressed on substance-use status, age, gender, and ISS.

The complications reported in the NTDB were used to test for the presence of significant differences in hospital course associated with pre-injury substance use. Complications positively associated with pre-injury substance use would suggest that hospital course is worse for substance-related trauma incidents than incidents without pre-injury substance use. The NTDB delimits several complication descriptions that cover a wide range of complications such as pneumonia, deep-venous thrombosis (DVT), cardiac arrest, and renal failure. Each complication was tested for its association with pre-injury substance use with logistic regression models in which the complication was modeled as a dichotomous response variable and regressed on substance-use status, age, gender, and ISS.

Pre-injury substance abuse may be associated with poorer outcomes following hospitalization. To simplify the outcomes analysis, the twelve different outcome descriptions contained in the NTDB were grouped into a more compact and less redundant classification scheme. The classification scheme is as follows: (i) NTDB outcomes "Death", "DOA", "Died during treatment" were grouped together as "death"; (ii) "Home", "Home Health", and "Jail" were grouped together as "home"; (iii) "Nursing home" and "SNF" were aggregated into "nursing home"; and (iv) "Rehab" remained a separate group. Incidents in which the description was either "Burn" or "Other" were not considered because of the difficulty in interpreting these outcomes. Each outcome was tested for its association with pre-injury substance use with Chi-Square tests on contingency tables in which both the specific outcome and substance-use status were modeled as dichotomous variables.

### Statistical Analysis

All statistical analyses were performed in R, version 2.8.1 (freely available from The R Project for Statistical Computing). Comparisons among groups were made using Wilcoxon-Mann-Whitney or Kruskal-Wallis rank sum tests. Categorical data were analyzed using Chi-square tests on contingency tables, or by using logistic regression models to estimate odds ratios for the risk associated with particular variables of interest. A value of *α *≤ *0.05 *was accepted as significant; when necessary, a Bonferroni-corrected value of *α *≤ *1*/*N *was used to assess significance, where *N *is the number of statistical tests.

An ANOVA model was used to test the significance of observed differences in complication rates among the four substance-use groups, while controlling for differences in age, gender, and injury severity (ISS). Tukey's "Honest Significant Differences" were computed to estimate confidence intervals on the coefficients of each substance-use group and control for the multiple hypothesis tests performed. The overall false-positive rate was maintained at α = 0.05.

Logistic regressions were carried out using the `lrm()` function in the 'Design' package (available from http://biostat.mc.vanderbilt.edu/twiki/bin/view/Main/Design) which is freely available from CRAN http://cran.r-project.org/. We used logistic regression modeling to estimate odds ratios for the association of pre-injury drug or alcohol use with (i) pre-existing comorbidities, (ii) complications arising during treatment, and (iii) outcomes following hospitalization. We state that a condition is *associated *with substance use when the estimated odds ratio was statistically significantly greater than one. Logistic regression models controlled for substance-use status, age, gender, and injury severity score (ISS). The fit of the regression model to the data was assessed using Chi-Square tests (α = 0.05); the full regression model was found to fit the data significantly well for all results presented herein.

## Results

### Demographics

Version 7.1 of the NTDB contains 1,926,244 patient records, of which 225,081 (11.6%) met our inclusion criteria (see Methods for details). The demographics of the patients are summarized in Table 1. The incidents in our dataset more frequently involved males (*n *= 162,227; 72%) than females (*n *= 62,854; 28%). There were significantly more males than females in the trauma incidents with pre-injury drug or alcohol use (Chi-square test, *P < 0.001*). Drugs and alcohol were significantly more likely to co-occur in the same incident than expected from their individual frequencies (Chi-square test, *P < 0.001*). The mean age of patients was 36.3 years for alcohol related incidents, 37.1 for drug related incidents, 34.3 for alcohol-and-drug related incidents, and 42.1 years for substance negative incidents, respectively (Kruskal-Wallis rank sum test, *P < 0.001*).

**Table 1:** Patient demographics.

	Alcohol Present	Drugs Present	Drugs and Alcohol Present	Neither Present
				
***Number of Incidents***	40,714 (18.1%)	58,353 (25.9%)	51,702 (23.0%)	74,312 (33.0%)
				
***Gender***				

Female	20.1%	29.8%	20.1%	36.2%
Male	79.9%	70.2%	79.9%	63.8%
				
***Age***				

16 - 20	11.5%	17.1%	12.8%	16.8%
21 - 30	31.8%	24.9%	32.5%	20.3%
31 - 40	20.5%	19.6%	23.4%	15.3%
41 - 50	19.4%	19.1%	20.6%	15.0%
51 - 60	10.2%	10.2%	7.7%	12.5%
61 - 70	4.0%	4.4%	2.0%	7.6%
71 - 80	2.1%	3.0%	0.8%	7.5%
81 - 90	0.6%	1.7%	0.3%	5.0%
				
***Mean ISS (SD)***	11.2 (10.9)	12.5 (10.9)	11.5 (10.6)	11.7 (11.2)

Pre-injury substance use has been proposed to cause more severe injuries, however prior studies have not consistently established such a relationship. Therefore, the ISS score for each incident was used to determine if drug or alcohol related trauma incidents were associated with more severe injuries. The median ISS score was 9 for all four classes of trauma incidents. The mean ISS scores were 11.2 for alcohol related, 12.5 for drug related, 11.5 for drug-and-alcohol related, and 11.7 for substance-negative trauma incidents, respectively (Kruskal-Wallis rank sum test, *P < 0.001*). Pairwise Wilcoxon-Mann-Whitney tests suggested that drug use is associated with more severe injuries than those experienced in substance negative incidents (*P << 0.001*), whereas alcohol is not (*P = 0.08*). Major trauma (defined as ISS ≥ 15) was found to occur in 28.0%, 31.4%, 28.5%, and 29.0% of the alcohol related, drug related, alcohol-and-drug related, and substance negative incidents, respectively (Chi-square test, *P < 0.001*).

### Pre-existing Comorbidities

Pre-injury drug or alcohol use may be associated with a less healthy subset of the overall population of trauma patients. We therefore determined whether patients involved in drug or alcohol related trauma visits are more likely to present to the ED with pre-existing comorbid conditions than patients involved in incidents where pre-injury substance use was not a factor. Specifically, logistic regression models were used the odds ratio for the association of each comorbid condition with substance-use status, while controlling for age, gender, and injury severity score (ISS).

There are 52 comorbid conditions reported in the NTDB, 44 of which were found to occur in the incidents included in this study. Eighty percent (35/44) of the reported comorbid conditions were not significantly associated with pre-injury drug or alcohol use. Six of the comorbid conditions were significantly associated with alcohol-related trauma visits, seven were significantly associated with drug related incidents, and 4 were significantly associated with alcohol-and-drug related visits (Table 2). Nineteen of the reported pre-existing comorbidities were significantly associated with trauma incidents with no pre-injury substance use. The top three, in decreasing order of association are pregnancy (O.R. = 5.4; P < 0.001), hemophilia (O.R. = 3.0, P = 0.002), and serum creatinine (2 mg) on admission (O.R. = 2.4; P = 0.02).

**Table 2:** Pre-existing comorbidities.

*A. Pre-existing comorbidities associated with alcohol-related trauma incidents*			
**Comorbidity (NTDB Description)**	***Number of Incidents***	**Odds Ratio**^**1**^	**(95% CI)**

Chronic Alcohol Abuse	5,455	9.72	(9.02,10.48)
Documented History of Cirrhosis	250	2.48	(1.89,3.26)
Acquired Coagulopathy	121	2.23	(1.48,3.31)
Chronic Pulmonary Condition	277	1.90	(1.45,2.49)
COPD	1,051	1.42	(1.22,1.65)
Chronic Drug Abuse	1,091	1.18	(1.05,1.34)
			
***B. Pre-existing comorbidities associated with drug-related incidents***			

**Comorbidity (NTDB Description)**	***Number of Incidents***	**Odds Ratio**	**(95% CI)**

Chronic Drug Abuse	5,079	7.80	(7.11,8.56)
HIV/AIDS	295	2.38	(1.83,3.11)
Organic Brain Syndrome	47	2.13	(1.17,3.89)
Chronic Alcohol Abuse	2,582	1.42	(1.30,1.54)
COPD	1,304	1.40	(1.24,1.58)
Documented History of Cirrhosis	235	1.34	(1.02,1.80)
History of Psychiatric Disorders	6,045	1.33	(1.26,1.41)
			
***C. Pre-existing comorbidities associated with alcohol-and-drug related incidents***			

**Comorbidity (NTDB Description)**	***Number of Incidents***	**Odds Ratio**	**(95% CI)**

Chronic Alcohol Abuse	6,839	7.35	(6.82,7.92)
Chronic Drug Abuse	5,004	7.65	(6.97,8.40)
Documented History of Cirrhosis	249	1.97	(1.46,2.65)
HIV/AIDS	250	1.99	(1.48,2.66)

**1**. All odds ratios are significant at the α = 0.01 level.

### Complications

Pre-injury drug and alcohol use may be associated with significantly worse hospital course, which was considered with two different approaches. To assess differences in the rate that complications arise during hospitalization, the mean number of complications per incident was compared across the four classes of trauma incidents. To determine whether specific complications were associated with pre-injury drug or alcohol use, logistic regression models were used to estimate odds ratios for the association between pre-injury substance use and each complication listed in the NTDB, controlling for age, gender, and injury severity score (ISS).

The vast majority of incidents - regardless of pre-injury drug or alcohol use - experienced no complications during treatment. The mean number of complications per incident was found to be 8.6, 11.3, 9.2, and 8.9 per one hundred alcohol related, drug related, alcohol-and-drug related, and substance negative trauma incidents, respectively. This corresponds to rates of complications arising during treatment of 8.3, 10.9, 9.9 and 8.6 per one hundred incidents in the alcohol related, drug related, alcohol-and-drug related, and substance-negative classes, respectively. Pre-injury drug use, is associated with small, but significant (ANOVA, *P << 0.001*) increases in the number of complications arising during treatment, but pre-injury alcohol use does not appear to be associated with significantly higher rates of complications during treatment.

Each complication listed in the NTDB was tested for its association with pre-injury substance use. Logistic regression models were separately fit to the alcohol related, drug related, and alcohol-and-drug related incidents to determine the effects of pre-injury alcohol use, pre-injury drug use, and pre-injury combined alcohol and drug use, respectively. Odds ratios were subsequently estimated from the logistic regression models for the substance-related risk associated with each complication, controlling for age, gender, and ISS.

There are a total of 24 unique complication descriptions delimited by the NTDB. Five were associated with pre-injury alcohol use, twelve were associated with pre-injury drug use, and 7 were associated with pre-injury use of both alcohol and drugs (Table 3). Infection-related complications were frequently associated with substance use: 80% of the alcohol associated complications, 50% of the drug associated complications, and 71% of the alcohol-and-drug associated complications were related to infection. Combining all infection-related complications together into a single response variable revealed that pre-injury drug and alcohol use is associated with 15% and 30% greater risk of infection, respectively (*P < 0.001*). In addition to infection, 42% (5/12) of the complications associated with pre-injury drug use were related to circulatory conditions (e.g. deep-venous thrombosis).

**Table 3:** Treatment complications.

*A. Treatment complications associated with alcohol-related visits*			
**Complication**	***Number of Incidents***	**Odds Ratio**^**1**^	**(95% CI)**

Aspiration Pneumonia	931	1.65	(1.45,1.89)
Intra-abdominal Abscess	168	1.38	(1.01,1.88)
Hypothermia	394	1.29	(1.05,1.59)
Wound Infection	1,112	1.15	(1.03,1.31)
Pneumonia	4,889	1.09	(1.02,1.15)
			
***B. Treatment complications associated with drug-related visits***			

**Complication**	***Number of Incidents***	**Odds Ratio**^**1**^	**(95% CI)**

Pulmonary Embolus	651	2.03	(1.73,2.40)
Wound Infection	1,112	2.01	(1.78,2.28)
Hypothermia	394	1.99	(1.62,2.46)
Skin Breakdown	683	1.77	(1.51,2.07)
Compartment Syndrome	663	1.66	(1.42,1.95)
Aspiration Pneumonia	931	1.59	(1.40,1.83)
Pneumonia	4,889	1.53	(1.44,1.62)
Pneumothorax	916	1.46	(1.28,1.67)
DVT (lower extremity)	1,619	1.42	(1.28,1.57)
Empyema	282	1.42	(1.12,1.81)
Urinary Tract Infection	3,060	1.28	(1.19,1.37)
Bacteremia	1,038	1.24	(1.09,1.40)
			
***C. Treatment complications associated with alcohol-and-drug related visits***			

**Complication**	***Number of Incidents***	**Odds Ratio**^**1**^	**(95% CI)**

Aspiration Pneumonia	931	1.76	(1.53,2.03)
Hypothermia	394	1.58	(1.27,1.97)
Intra-abdominal Abscess	168	1.59	(1.15,2.20)
Pneumonia	4,889	1.22	(1.14,1.30)
Pneumothorax	916	1.36	(1.18,1.58)
Skin Breakdown	683	1.32	(1.10,1.58)
Wound Infection	1,112	1.45	(1.27,1.66)

1. All odds ratios are significant at the α = 0.01 level.

Chronic drug and alcohol use may be associated with patients experiencing a different set of complications than those experienced by acute users. This hypothesis was addressed by removing 18,451 (8.2%) incidents in which the patient was reported to have either "chronic alcohol abuse" or "chronic drug abuse" as a pre-existing comorbidity. The logistic regression models were subsequently reconstructed and revised odds ratios were estimated. The results shown in Table 3 were qualitatively unchanged (not shown). Acute pre-injury drug or alcohol use therefore appeared to be associated with increased risk of developing infection-related complications during the course of treatment.

Pre-injury substance use may be associated with significantly greater hospital charges. We considered this hypothesis by comparing the mean charges among the different classes of trauma incidents. Unfortunately, 135,802 (60.3%) of the incidents in our dataset did not have a valid charge entry (e.g. 120,333 incidents had a negative or zero charge). Using the remaining 89,279 incidents, the mean charges were $60,407.90 for alcohol-related trauma incidents, $67,991.82 for drug-related trauma incidents, $96,739.45 for alcohol-and-drug-related incidents, and $54,981.23 for substance-negative incidents. Pre-injury drug and alcohol use is associated with significantly greater hospital charges for the treatment of trauma patients (Kruskal-Wallis rank sum test, *P << 0.001*). The complications linked to pre-injury drug or alcohol use were associated with significantly larger hospital charges (Table 4). The amount of increase averaged in the tens of thousands of dollars, and was several fold more than treating an incident without complications. The increased charges partly result from the fact that these complications are associated with significantly longer lengths of stay, often by more than fourteen days, relative to an incident without complications (Table 4).

**Table 4:** Increased hospital charges and lengths of stay for treatment complications.

Complication	Incremental Increase in Charges		Incremental Increase in Length of Stay	
	**Dollars (US$)**	**Fold Increase**^**1**^	**Days**	**Fold Increase**
	
Renal Failure	$60,174.22	4.8	24.0	5.8
Bacteremia	$58,776.73	4.7	24.8	6.0
Pneumonia	$52,442.26	4.3	22.2	5.5
Intra-Abdominal Abscess	$50,726.11	4.2	33.0	7.6
Compartment Syndrome	$37,169.68	3.4	13.5	3.7
Skin Breakdown	$34,284.75	3.2	32.6	7.6
Wound Infection	$32,092.39	3.0	26.5	6.3
Pneumothorax	$31,812.20	3.0	16.3	4.3
Hypothermia	$27,505.99	2.7	7.1	2.4
DVT (Lower Extremity)	$24,554.94	2.6	23.7	5.8
Pulmonary Embolus	$23,806.46	2.5	17.0	4.4
Aspiration Pneumonia	$16,814.81	2.1	15.0	4.0
Esophageal Intubation	$10,304.99	1.7	12.5	3.5
				
**All infection-related**	**$43,809.12**	**3.8**	**21.0**	**5.2**
**All vascular-related**	**$27,463.63**	**2.7**	**17.8**	**4.6**

1. Fold increase is calculated by dividing the mean cost of an incident in which the complication arose by the mean cost of an incident in which no complications occurred.

### Outcomes

Discharge information contained in the NTDB was used to assess whether pre-injury substance use is associated with significantly worse clinical outcomes. The NTDB discharge descriptions were grouped to simply the analysis (see Methods for details). Discharge to "Home" occurred most frequently in the alcohol and drug related incidents, occurring in 73.4%, 70.5%, 72.0, 70.0% of the alcohol related, drug related, alcohol-and-drug related, and substance negative trauma visits, respectively (Chi-square test; P < 0.001). The increased association of "Home" with alcohol and drug use is partly caused by discharge to "Jail" (a component of "Home"), which is 1.8 times more likely to occur in the population of drug and alcohol users (Chi-square, P << 0.001). Discharge to "nursing home" occurred more frequently in the substance-negative incidents (4.8%) than the alcohol related (2.5%), drug related (4.0%), and alcohol-and-drug related (2.9%) incidents (Chi-square test; P << 0.001). A similar trend was observed for discharge to "rehab", which occurred in 8.1%, 9.5%, 7.3%, and 10.8% of the alcohol related, drug related, alcohol-and-drug related, and substance-negative incidents, respectively (Chi-square test; P < 0.001). "Death" occurred in 3.6%, 3.4%, 3.0%, 4.5% of the alcohol related, drug related, alcohol-and-drug related, and substance-negative incidents, respectively (Chi-square test; P < 0.001).

## Discussion

While advances such as the development of specialized trauma centers have helped to decrease mortality by as much as 20%, the health and economic costs associated with traumatic injury remain staggering. Traumatic injury is the leading cause of death for Americans under the age of 45 and the fifth most common cause of death for the nation as a whole [2]. It has been estimated that one-third of all intensive care admissions are related to trauma, and medical spending on trauma care ranks second; only cardiac disease commands more of the nation's healthcare dollars. In light of this, any effort that will improve care delivery or decrease costs could have a major impact on the healthcare system.

Drug and alcohol use has been shown to have a strong association with traumatic injury. Between 30% and 40% of trauma patients test positive for drug use and 27% to 47% of patients test positive for alcohol use at the time of admission [8]. In an effort to improve care, many studies have focused on implementing strategies such as counseling for this patient population, whereas fewer have examined the effects of drug and alcohol use on patients' medical care and outcome following a traumatic injury [5,6,9,14,17,36-39].

In the present study, we used the National Trauma Data Bank to determine the whether pre-injury alcohol or drug use is associated with (i) patient health prior to injury, (ii) injury severity, (iii) hospital course following injury, and (iv) outcome following hospitalization. In general, patients involved in drug or alcohol related trauma incidents do not appear to be appreciably less healthy prior to injury than the trauma population at large. The majority (80%) of the comorbid conditions were found to occur at indistinguishable frequencies among the four classes of trauma incidents. Furthermore, many of the comorbid conditions associated with pre injury alcohol and drug use, such as "Chronic Alcohol Abuse", "Chronic Drug Abuse", and "Documented History of Cirrhosis", are expected to be overrepresented in this population of patients.

Pre-injury alcohol or drug use was not associated with more severe injures than incidents where substance use was not a factor. The median ISS score was the same for all four classes of trauma incidents, and only slight differences were observed in the mean ISS score among groups. Drug use (not in conjunction with alcohol) may be associated with slightly higher frequencies of major trauma (ISS ≥ 15). Pre-injury drug use may therefore be associated with small, but significant, increases in injury severity, whereas pre-injury alcohol use is not.

Substance use does not appear to have a dramatic adverse effect on patient outcomes following hospitalization, which is consistent with a recent study finding that intoxicated drivers may have more favorable outcomes and reduced mortality [40]. The drug and alcohol related visits were associated with significantly more frequent discharge to home, which was partly the result of significantly more frequent discharge to jail in this population. In contrast, discharge to "rehab", "nursing home", or "death" occurred significantly more frequently in the substance-negative visits. Drug and alcohol related trauma incidents were therefore followed by generally better outcomes than substance negative incidents, which may be partly related to age. The substance-negative incidents were found to occur in a significantly older population, which would be expected to have more difficulties overcoming traumatic injury.

Though pre-injury alcohol and drug use was associated with neither less-healthy patients nor worse patient outcomes, important differences were observed with respect to treatment course, and these differences were found to be associated with pre-injury drug and alcohol use. Patients testing positive for alcohol or drugs developed infection-related complications while hospitalized at a significantly higher rate than those testing negative (Table 3). In addition, patients testing positive for drugs were found to be at significant risk to develop additional complications related to the vascular system, such as deep venous thrombosis. Pre-injury use of both drugs and alcohol prior to injury did appear not to be associated with significantly worse hospital course than use of these substances individually.

The deleterious effects of alcohol use on the body's ability to combat infection after trauma include respiratory depression and an impaired cardiovascular response to injury [1,4,29,41,42]. In addition, alcohol dampens the body's release of catecholamines following injury, thereby lowering tissue oxygen delivery [20]. The cytotoxic effects of alcohol on gastric epithelium and the hepatocytes may also increase patients' susceptibility to infection [29]. Alcohol is also known to be directly immunosuppressive, an effect that may last as long as seven days [1,4]. Polymorphonuclear neutrophil and T-lymphocyte function is impaired by alcohol ingestion as is the production of key cytokines for fighting infection such as tumor necrosis factor alpha and interleukin 8.

The link between drug use and infectious complications after trauma can also be explained by an impaired respiratory and cardiovascular response to injury [43]. Previous studies have shown that patients using cocaine and sedatives are more likely to require mechanical ventilation during hospitalization [44]. A recent investigation of methamphetamine use in trauma patients found that users were 62% more likely to require ventilator support following injury [45].

The findings in this study illustrate that alcohol and drug use has appreciable effects on both biological and economic aspects of patient care. The hospital charges associated with drug and alcohol related trauma incidents were significantly greater than those for substance negative incidents. We noted that a large number of cases did not contain valid charge information and were therefore excluded from the analysis. We caution that there may be some bias in the cases included in this particular analysis. The length of hospitalization was also found to be longer in the drug and alcohol related trauma incidents. This may, in part, be related to the greater association of infection complications with pre-injury drug and alcohol use because incidents in which infection complications resulted in significantly greater hospital charges.

For example, knowledge of a patient's drug and alcohol use could help trauma care providers manage patients[15,22,36]. Prophylactic measures, such as surveillance for infections, could be taken to lower the infectious complication rate in patients presenting to the ED with alcohol or drugs in their blood. This could lead to shorter hospitalizations and lower treatment costs. Unfortunately, even though providers recognize the importance of an admission toxicology screen, there is a growing legal impediment to testing patients for drug or alcohol intoxication (46). The Uniform Accident and Sickness Policy Provision Law (UPPL) is a legal statute permitting insurers to deny coverage to patients who are injured while under the influence of alcohol or drugs. One recent study found that over a six-month period, 24% of surveyed providers stated that they had been denied payment under this statute for care they delivered (46). More than 90% stated that they believed that toxicology screening was important and many cited the UPPL as a reason that they have changed their practice and no longer screen patients.

For example, knowledge of a patient's drug and alcohol use could help trauma care providers manage patients[15,22,36]. Prophylactic measures, such as surveillance for infections, could be taken to lower the infectious complication rate in patients presenting to the ED with alcohol or drugs in their blood. This could lead to shorter hospitalizations and lower treatment costs. Unfortunately, even though providers recognize the importance of an admission toxicology screen, there is a growing legal impediment to testing patients for drug or alcohol intoxication (46). The Uniform Accident and Sickness Policy Provision Law (UPPL) is a legal statute permitting insurers to deny coverage to patients who are injured while under the influence of alcohol or drugs. One recent study found that over a six-month period, 24% of surveyed providers stated that they had been denied payment under this statute for care they delivered (46). More than 90% stated that they believed that toxicology screening was important and many cited the UPPL as a reason that they have changed their practice and no longer screen patients.

There are some caveats to this study that should be considered. The NTDB is a database of voluntary submissions by contributing hospitals, and thus cannot be considered a representative sample of trauma incidents. We attempted to minimize biases inherent in the database as much as possible. The results obtained herein used incidents from 490/770 (64%) of the contributing hospitals, including 81/101 (80%) of the ACS level-1 trauma centers and all four geographic regions. The quality of the NTDB data is subject to the efforts of the contributing hospitals. To overcome this limitation, we strictly checked our data to make sure that the fields of interest contained reasonable values. Another data-quality limitation is evident in the 'ALCOHOLPRE' and 'DRUGSPRE' variables, which only capture the presence or absence of drugs or alcohol, and not the quantity present. Our final dataset therefore likely contains patients with varying amounts of these substances in their bodies at the time of injury. Additionally, the 'DRUGSPRE' variable only indicates if "controlled substances other than alcohol" were detected in the patient's blood or urine; the definition specifically excludes drugs administered "during any phase of resuscitation". Unfortunately, what constitutes a controlled substance is left to the discretion of the reporting hospital, and thus, while the 'DRUGSPRE' variable is intended to capture illegal drug use (e.g. cocaine or heroine), some of the incidents studied herein may not be related to the pre injury use of such substances (S. Goble, personal communication). Voluntary controlled-substance testing and reporting also means that some patients in the substance-negative category may actually presented to the ED with alcohol or drugs in their blood at the time of injury. It's unclear the impact of such substance negative patients on our results, however our results may be conservative if such patients experienced more complications that most truly substance-negative patients.

Alcohol and drug use have significant negative health effects that are magnified following trauma. Pre-injury alcohol and drug use was found to be associated with significant risk for developing infection or circulatory complications during hospitalization. The charges associated with treating these incidents were found to be significantly greater than incidents in which drugs or alcohol was not a factor. Every effort must therefore be made to encourage screening at the time of admission so practitioners can treat patients as effectively as possible. This may mean that practitioners will have to work to overturn hindering legislation such as the UPPL.

## Competing interests

The authors declare that they have no competing interests.

## Authors' contributions

MC participated in the design of the studies, performed the biostatistical analysis, interpreted the data and drafted the manuscript. MB participated in the design of the studies, assisted with the data analysis and drafted the manuscript. Both authors read and approved the final manuscript.
